# Growth, carcass parameters, biochemical and oxidative stress indices, and meat traits of duck breeds under different stocking densities

**DOI:** 10.1016/j.psj.2022.101992

**Published:** 2022-06-05

**Authors:** Mohammed A.F. Nasr, Adel Q. Alkhedaide, Marwa M.E. Radwan, Abd-El Salam E. Hafez, Mohamed A. Hussein, Rasha M. El Bayomi

**Affiliations:** ⁎ Animal Wealth Development Department, Faculty of Veterinary Medicine, Zagazig University, Zagazig 44511 Egypt; † Department of Clinical Laboratory Sciences, Turabah University College, Taif University, Taif 21944, Saudi Arabia; ‡ Food Control Department, Faculty of Veterinary Medicine, Zagazig University, Zagazig 44511, Egypt

**Keywords:** stocking density, muscovy duck, Mallard duck, performance, biochemical parameters

## Abstract

This investigation was aimed to inspect if there is an influence of various stocking density on growth, carcass parameters, blood indices, and meat traits of Muscovy and Mallard ducks. One hundred twenty-six 1-day-old of each Muscovy and Mallard ducks were randomly allocated into three experimental groups with different stocking density. Group one (**SD_1_**) was 5 ducks/m^2^, while group 2 (**SD_2_**) was 7 ducks/m^2^ and group 3 (**SD_3_**) was 9 ducks/m^2^. The growth, carcass parameters, meat quality, blood indices were calculated. Body weight of SD_1_ was 18 and 4.5% heavier than SD_2_, while, it was 29.5 and 12% heavier than SD_3_ of Muscovy and Mallard duck breeds, respectively. SD_3_ possessed the highest levels of, H/L, ALT, AST, LDL, VLDL, and MDA with the lowest levels of lymphocyte, SOD,GSH, GPX, C3, total antioxidant capacity and IGG of both ducks' breeds. The carcass weight decreased by 40 and 15% from SD_1_ to SD_3_ in Muscovy and mallard ducks, respectively. The dressing % was highest at SD_1_ (84 and 83%) when compared with SD_3_(71and80%) of Muscovy and Mallard ducks, respectively. Cooking loss was 20 and 16% greater in group three when compared with group one in Muscovy and Mallard ducks, respectively. In conclusion ducks raised in low SD possessed the best performance with better welfare.

## INTRODUCTION

In the last decade the duck meat (*Anas platyrhynchos domestica*) necessity has obviously greater than before. It is incredibly consumed in lots of countries, such as United States, Egypt, and Saudi Arabia (Wawro et al., 2004). Egypt produced around 100 to 150 million domestic ducks annually, therefore, it is considered “duck country” (Kilany et al., 2016), and it plays a chief role in the rural economy and creates employment opportunity. Ducks are characterized by rapid growth especially at the first weeks of age. Muscovy and Mullard ducks reach the slaughter age at 10 to 12 and 10 wk, respectively with weights represented 70 to 80% of adult weight (Pingel, 1999). Ducks are more tolerance to tough climates when compared with chicken, therefore, their rearing are easier for producers (Ekarius, 2007; Anonymous, 2016) due to the less likely susceptible to most common poultry diseases (i.e., Marek's and infectious bronchitis; Oluyemi and Ologbobo, 1997).

Duck meat is considered high nutritive value and favorable meat for consumers (Taboosha, 2014). It has 1) polyunsaturated fatty acids, 2) a promising amino acid content (Woloszyn et al., 2006; Ali et al., 2007), 3) high protein and ash %, 4) low fat and water %, 5) high % of red muscle fiber in breast meat, therefore it may be called as red meat (Ali et al., 2007). Furthermore, its liver has a crucial role of income for farmers and is mostly collected from the hybrids of Pekin and Muscovy ducks (Holderread, 2011). In addition to, Mullards were mainly used for producing fattened liver, but recently used for broiler production as 35% of duck meat in France originated from Mullards (Baeza et al., 2000).

The common ducks used for meat production are Pekin, Aylesbury, Rouen, and Muscovy ducks (Crawford, 1990; Pingel, 2004). Muscovy ducks originate from domestication of the mallard duck (*Anas platyrhynchos*) (Cherry and Morris, 2015). Muscovy ducks were very popular based on their adaptation to rearing environment with low fat and high meat production (Górski and Witak, 2003). Recently, meat duck production reared under intensive system, therefore, lots of studies are required to afford appropriate animal welfare circumstances with producing high meat quality (Chen et al., 2015). The suggested stocking density of the duck farms in Egypt is 5 ducks/m^2^ for higher growth performance (Taboosha, 2014). In the last decade consumers considered stocking density as it may be influenced animal wellbeing (Vanhonacker et al., 2008).

Stocking density (**SD**) is distinct as birds' number of birds reared in specific area (Berg and Yngvesson, 2012). Fundamental intentions of worldwide poultry business are intensifying poultry meat production (kg) per m^2^ with top-quality and to put off production losses initiated by excess numbers. Poultry high SD is mainly intended to reduce the cost interrelated with employment, gas, sheltering, and tools. However, this can lead to a negative consequence on birds wellbeing, immune status, welfare, and production traits (Shanawany, 1988; Houshmand et al., 2012). Birds are covered with feathers and do not possess sweat glands, therefore they are struggling at high temperatures especially ≤41˚C and high SD that consequently may break homeostasis (Etches et al., 2008; Mello et al., 2015). Therefore, this study was aimed to explore the influence of various SD on growth traits, carcass parameters, blood indices, and meat quality of Muscovy and Mallard ducks to advocate good SD alongside cheapest coast concurrently with superior property.

## MATERIALS AND METHODS

### Birds and Management

The current research was done at Faculty of Veterinary Medicine Zagazig University, Egypt. One hundred twenty-six 1-day old Muscovy and Mallard ducks were randomly classified to three groups each of 6 replicates. The first one (**SD_1_**) was 5 ducks/m^2^. The second (**SD_2_**) was 7 ducks/m^2^. The third (**SD_3_**) was 9 ducks/m^2^. Ducks were reared in similar pens with temperature of 32˚C for the first 7 d of age. The temperature was regularly reduced to 24˚C at the age of 21 d and continued to the 10th wk. Ducks were fed ad libitum (NRC, 1994; Table 1).

**Table 1:** Chemical composition of basal diets.

	Starter diet	Grower diet
Ingredients (%)	One-day-old–4 wk of age	5–10 wk of age
Ground yellow corn	54.14	65.05
Soybean meal (44 %)	40.10	29.19
Soyabean oil	2.90	2.90
Limestone	1	1
Di- calcium phosphate	1	1
Methionine	0.11	0.11
Nacl	0.25	0.25
Premix^1^	0.50	0.50
Calculated analysis		
Crude protein %	22.00	18.01
Crude fiber %	3.72	4.44
Metabolizable energy, kcal ME /kg	2,945	2,985
Ash	7.69	6.17
Dry matter	92.50	93.07
Ether extract	3.77	3.36

1 The premix provided each kg of diet with: Vit. A: 12,000 IU, Vit. D3: 5,000 IU, Vit. E: 130 mg, Vit. K3: 3.6 mg, Vit. B1: 3 mg, Vit. B2: 8 mg, Vit. B6: 4.95 mg, Vit. B12: 0.17 mg, Niacin: 60 mg, Folic acid: 2.10 mg, d-Biotin: 200 mg, calcium d-Pantothenate: 18.3 mg, Copper: 80 mg, Iodine: 2 mg, Selenium: 150 mg, Iron: 80 mg, Manganese: 100 mg, Zinc: 80 mg, Cobalt: 500 mg.

### Growth Traits and Blood Profile

Ducks were weighed bi-weekly for obtained, body weight, daily gain, and relative growth rate. Blood samples were collected immediately of early disturbance at the day of slaughtering (3, 4, and 5 blood samples from SD_1_, SD_2_, and SD_3_, respectively from each pen of each replicate). Two blood samples were smoothly obtained from the wing vein under aseptic circumstances. The first one with anticoagulant was centrifuged at 4,000 rpm for 15 min to gather the serum. Total protein, albumin, aspartate transaminase (**AST**), alanine transaminase (**ALT**), creatinine, urea, triglyceride (**TG**), high-density lipoprotein (**HDL**) cholesterol (mg/dL), low-density lipoprotein (**LDL**), and very low-density lipoprotein (**VLDL**) values were evaluated with commercial kits (Wako Pure Chemical Industries, Osaka, Japan). Superoxide dismutase (**SOD**) and glutathione peroxidase (**GSH**), malondialdehyde (**MDA**), and reduced glutathione (**GSH**) values were evaluated using commercial kits (Biomericux, Poains, France) and a spectrophotometer (Shimadzu, Japan). Complement C and immunoglobulin G (**IgG**) values were evaluated using Chicken Immunoglobulin G (IgG) ELISA Kit (Cat No. MBS260043) with sensitivity up to 5 ng/mL, intra-assay precision <= 8% and inter-assay precision ≤ 12%. While, the second samples without anticoagulant were used to evaluate lymphocyte and heterophil.

### Carcass Properties

At the 10th wk of the investigation, ducks were weighed and slaughtered. The gizzard, heart, liver, and spleen were collected, weighed. They offered as a relative weight. The carcass (breast and thigh) was weighed and the dressing % was calculated.

### Meat Parameters

Ten grams of Longissimus thoracis et lumborum (**LTL**) muscles were collected from the chilled carcass to evaluate pH, cooking loss, drip loss, and bacteriological counts. Ultimate pH (**pHu**) was measured after 24 h of chilling (Korkeala et al., 1986). Cooking loss was evaluated (Cyril et al., 1996). Drip loss was evaluated by the weight variation between frozen and thawing meat and blotting dry with filter paper (for more details see; Nasr et al., 2021). Total bacterial count, Enterobacteriaceae and Coliforms were counted (cfu/g) (for more details see; Nasr et al., 2021).

### Statistical Analysis

Result was statistically analyzed with SAS statistical system Package V9.1 (SAS. 2009). Kolmogorov–Smirnov test was carried out to assure the homogeneity and normality of variances among the various investigating groups. Ducklings were distributed according to a completely randomized experimental design in an ANOVA of 2 × 3 factorial arrangement. The statistical model used was:

$$Y_{\text{ijk}}=\mu +SD_{i}+B_{j}+\text{SD}B_{\text{ij}}+e_{\text{ijk}}$$

where: Y_ijk_ = an observation, µ = overall mean, SD_i_ = stocking density levels (i = 5, 7, and 9 birds), B_j_ = breed (j = Muscovy and Mallard), SDB_ij_ = the interaction between SD and breed and e_ijk_ = random error. The variation between means was performed with Tukey's test. Log geometric mean was calculated for bacteriological counts. The significant was considered at *P* < 0.05.

## RESULTS

Body weight was significantly affected by SD. The final body weight of SD_1_was the highest during the whole rearing period of both ducks' breeds. It was 18% (*P* = 0.000) and 4.5% (*P* = 0.000) heavier than SD_2_ of Muscovy and Mallard breeds, respectively. While, it was 29.5% (*P* = 0.000) and 12% (*P* = 0.000) heavier than SD_3_ of Muscovy and Mallard breeds, respectively. The average daily was the highest at SD_1_ of both ducks' breeds when compared with SD_2_ and SD_3_ (Table 2). SD did not affect the levels of urea, total protein, albumin, globulin, and A/G ratio. But the variation was significant regarding lymphocyte, heterophil, H/L ratio, ALT, and AST of both ducks' breeds. Heterophil, H/L ratio, ALT, and AST revealed the highest values at SD_3_ of both breeds, while lymphocyte was the lowest (Table 3).

**Table 2:** Growth performance of ducks reared with different stocking density.

	Muscovy breed					Mallard breed				
Parameters	SD_1_	SD_2_	SD_3_	SEM	*P-*value	SD_1_	SD_2_	SD_3_	SEM	*P*-value
Body weight (g) at different weeks of age										
Day one	51.56	51.90	51.87	0.09	0.31	57.32	57.20	57.31	0.07	0.75
2^nd^ week	246.20^a^	240.96^b^	241.94^b^	0.64	0.002	255.58^a^	248.34^b^	247.12^b^	1.01	0.000
4^th^ week	926.00^a^	825.00^b^	798.33^b^	14.27	0.000	509.00^a^	471.43^b^	422.11^c^	10.20	0.000
6^th^ week	2082.00^a^	1869.29^b^	1705.00^c^	36.88	0.000	1902.00^a^	1727.86^b^	1453.89^c^	46.62	0.000
8^th^ week	3029.00^a^	2541.43^b^	2287.22^c^	67.06	0.000	3149.60 a	3017.14^a^	2738.22^b^	54.90	0.002
10^th^ weeks	3882.00^a^	3197.14^b^	2737.78^c^	104.29	0.000	4114.00 a	3929.71^b^	3631.44^c^	47.55	0.000
Average daily gain (g/d) at different weeks of age										
0-1 week	89.03^a^	87.60^b^	86.91^b^	0.27	0.003	89.96	89.67	88.88	0.48	0.65
2-3 week	123.80	76.89	100.84	9.85	0.209	101.66 b	104.50^a^	86.45^c^	1.95	0.000
4-5 week	602.00^a^	519.29^b^	481.11^b^	16.24	0.007	656.36	647.67	631.44	9.55	0.58
6-7 week	532.00^a^	353.57^b^	296.11^b^	28.63	0.001	580.00 b	675.71^ab^	776.67^a^	31.65	0.04
8-9 week	504.00^a^	263.57^b^	213.89^b^	32.20	0.000	356.40	371.28	547.22	42.15	0.09
0-10 weeks	3830.44^a^	3145.24^b^	2685.90^c^	104.32	0.000	4056.68 a	3872.52^b^	3574.13^c^	47.56	0.000
Relative growth rate (%) at different weeks of age										
0-1 week	172.68^a^	168.80^b^	167.57^b^	0.72	0.010	156.95	156.79	155.09	0.92	0.66
2-3 week	50.27	31.82	41.79	4.04	0.236	39.79^b^	42.09^a^	34.99^c^	0.75	0.000
4-5 week	65.35	63.28	60.66	2.03	0.675	130.49	138.26	149.95	3.86	0.12
6-7 week	25.65^a^	19.05^b^	17.49^b^	1.37	0.051	30.49^b^	39.29^b^	54.30^a^	3.14	0.002
8-9 week	16.65^a^	9.87^b^	9.42^b^	1.00	0.005	11.32	12.33	20.46	1.92	0.08
9-10 week	9.92	14.67	9.45	1.17	0.122	17.35^a^	15.97^a^	10.53^b^	0.85	0.000

SD_1_: stocking density of 5 ducks/m^2^; SD_2_: stocking density of 7 ducks/m^2^; SD_3_: stocking density of 9 ducks/m^2^.

a,b,c Means within the same row with different superscripts letter was differ significantly.

**Table 3:** Hematology, kidney, and liver parameters of ducks reared with different stocking density.

	Muscovy breed					Mallard breed				
Parameters	SD_1_	SD_2_	SD_3_	SEM^1^	*P-value*	SD_1_	SD_2_	SD_3_	SEM	*P-value*
Lymphocyte %	63.10^a^	59.76^b^	56.14^c^	0.65	0,000	62.00^a^	57.86^b^	52.67^c^	0.90	0.000
Heterophil %	24.96^c^	30.16^b^	33.73^a^	0.79	0,000	26.40^c^	31.71^b^	34.78^a^	0.79	0.000
H/L^2^	39.58^c^	50.48^b^	60.09^a^	1.85	0,000	42.63^c^	54.82^b^	66.09^a^	2.17	0.000
Creatinin (mg/dL)	1.00^a^	1.09^a^^b^	0.93^b^	0.03	0,032	0.93	0.93	0.95	0.01	0.78
Urea (mg/dl)	24.56	24.11	24.23	0.31	0,869	12.40	12.31	11.78	0.20	0.39
TP (g/dL)^3^	6.24	5.89	5.80	0.09	0,125	8.46	8.57	8.44	0.06	0.63
Albumin (g/dL)	3.81	3.71	3.58	0.10	0,676	5.80	5.88	5.89	0.07	0.89
Globulin (g/dL)	2.43	2.18	2.22	0.13	0,765	2.66	2.69	2.56	0.09	0.82
A/G^4^	1.77	1.75	1.82	0.15	0,982	2.18	2.24	2.48	0.15	0.69
ALT(U/L)^5^	15.27^b^	18.03^b^	17.17^ab^	0.42	0,039	24.38	26.41^c^	29.92^b^	0.56^a^	0.000
AST(U/L)^6^	21.58^b^	22.57^a^^b^	23.62^a^	0.31	0,020	19.78	24.66^c^	33.13^b^	1.25^a^	0.000

SD_1_: stocking density of 5 ducks/m^2^; SD_2_: stocking density of 7 ducks/m^2^; SD_3_: stocking density of 9 ducks/m^2^.

a,b,c Means within the same row with different superscripts letter was differ significantly.

1 SEM, standard error mean.

2 H/L, Heterophil/Lymphocyte ratio.

3 TP, total protein.

4 A/G, albumin/globulin ratio.

5 ALT, alanine transaminase.

6 AST, aspartate transaminase.

Cholesterol, triglyceride and HDL were not affected by different SD of Muscovy ducks, while they increased from SD_1_ to SD_3_ of Mallard ducks. SD_3_ possessed the highest levels of LDL, VLDL, and MDA with the lowest levels of SOD, GSH, GPX, C3, total antioxidant capacity, and IGG of both ducks breeds (Table 4). Carcass traits were significantly affected with different SD. The carcass weight decreased by 40% (*P* = 0.000) and 15% (*P* = 0.000) from SD_1_ to SD_3_ in Muscovy and mallard ducks, respectively. The dressing % was highest at SD_1_ (84 and 83%) (*P* = 0.000) when compared with SD_3_ (71 and 80%; *P* = 0.000) of Muscovy and Mallard ducks, respectively. SD_1_ was possessed the best breast and thigh weight of both breeds. By increasing SD, increased heart and spleen weight of both breeds (Table 5).

**Table 4:** Biochemical, oxidative stress, and immunological parameters of ducks reared with different stocking density.

	Muscovy breed					Mallard breed				
Parameters	SD_1_	SD_2_	SD_3_	SEM^1^	*P-*value	SD_1_	SD_2_	SD_3_	SEM	*P-*value
Cholesterol (mg/dL)	142.00	137.86	151.78	3.34	0.179	292.60^c^	333.14^b^	384.21^a^	8.51	0.000
Triglyceride (mg/dL)	135.60	134.00	163.44	7.26	0.146	60.12^c^	83.57^b^	154.00^a^	9.33	0.000
HDL (mg/dL)^2^	38.60	34.57	38.00	0.79	0.084	63.00^a^	56.43^b^	34.44^c^	2.85	0.000
LDL (mg/dL)^3^	68.92^b^	68.74^b^	114.38^a^	7.01	0.001	265.08^c^	283.83^b^	311.24^a^	4.38	0.000
VLDL (mg/dL)^4^	25.18^b^	29.44^b^	42.08^a^	1.93	0.000	17.08^c^	22.24^b^	32.96^a^	1.53	0.000
SOD (U/mL)^5^	6.76^a^	6.48^a^	5.23^b^	0.22	0.003	5.06	3.96	4.48	0.86	0.90
GSH (mmol/mL)^6^	2.71^a^	2.14^b^	1.79^c^	0.09	0.000	7.86^a^	5.01^b^	3.63^c^	0.38	0.000
GPX(U/mL)^7^	137.28^a^	132.91^b^	124.29^c^	1.25	0.000	144.20^a^	124.10^b^	103.49^c^	3.82	0.000
MDA(nmol/L)^8^	18.50^b^	20.70^b^	23.16^a^	0.61	0.003	27.96^c^	39.94^b^	79.60^a^	5.11	0.000
Total antioxidant capacity (mM/L)	1.60^a^	1.15^a^	0.63^a^	0.09	0.000	6.740^a^	4.18^b^	2.69^c^	0.37	0.000
C_3_ (ug/mL)^9^	1.30^a^	1.24^a^	0.64^b^	0.09	0.000	7.38^a^	5.96^b^	3.12^c^	0.40	0.000
IGG (ng/mL)^10^	3.73^a^	3.42^a^	2.59^b^	0.13	0.000	18.67^a^	11.93^b^	8.43^c^	0.96	0.000

SD_1_: stocking density of 5 ducks/m^2^; SD_2_: stocking density of 7 ducks/m^2^; SD_3_: stocking density of 9 ducks/m^2^.

a,b,c Means within the same row with different superscripts letter was differ significantly.

1 SEM, standard error mean.

2 HDL, high-density lipoprotein.

3 LDL, low-density lipoprotein.

4 VLDL, very low-density lipoprotein.

5 SOD, superoxide dismutase.

6 GSH, reduced glutathione.

7 GPX, glutathione peroxidase.

8 MDA, malondialdehyde.

9 C_3_, complement 3.

10 IgG, immunoglobulin G.

**Table 5:** Carcass traits and relative organs of ducks reared with different stocking density.

	Muscovy breed					Mallard breed				
Parameters	SD_1_	SD_2_	SD_3_	SEM^1^	*P-*value	SD_1_	SD_2_	SD_3_	SEM	*P-*value
Carcass weight (g)	3253.60^a^	2484.29^b^	1958.89^c^	117.84	0.000	3407.40^a^	3278.01^b^	2908.81^c^	50.78	0.000
Dressing %	83.81^a^	77.62^b^	71.49^c^	1.18	0.000	82.82^a^	83.41^a^	80.09^b^	0.37	0.000
Breast weight	1637.96^a^	1303.20^b^	1081.29^c^	50.32	0.000	1808.60^a^	1717.06^b^	1592.82^c^	23.17	0.000
Thigh weight	1414.40^a^	1006.66^b^	737.06^c^	62.19	0.000	1412.80^a^	1380.97^a^	1155.75^b^	27.46	0.000
Liver %	2.64	2.71	2.94	0.15	0.708	2.08	2.06	2.02	0.03	0.78
Heart %	0.76^c^	0.96^b^	1.12^a^	0.04	0.000	0.74^c^	0.77^b^	0.86^a^	0.01	0.000
Spleen %	0.15^c^	0.20^b^	0.26^a^	0.01	0.000	0.15^b^	0.15^b^	0.17^a^	0.001	0.000
Gizzard %	2.63	3.17	3.32	0.08	0.000	2.50	2.51	2.46	0.03	0.80

SD_1_: stocking density of 5 ducks/m^2^; SD_2_: stocking density of 7 ducks/m^2^; SD_3_: stocking density of 9 ducks/m^2^.

a,b,c Means within the same row with different superscripts letter was differ significantly.

1 SEM, standard error mean.

There was a significant effect of SD on meat quality of pectoral major muscles. Phu was the highest at SD_3_ of both breeds. By increasing SD, increased cooking loss of both breeds. It was 20 and 16% (*P* = 0.000) greater in stocking density of group three than group one of Muscovy and Mallard ducks, respectively. However, it was 17 and 8% (*P* = 0.000) higher when compared with SD_2_ in Muscovy and Mallard ducks, respectively. Moreover, the drip loss % was increased by increasing SD. The highest loss was recorded for SD_3_ in both breeds. The total bacterial counts, Enterobacteriaceae and Coliform bacteria were lowest in SD_1_ and SD_2_ of Muscovy ducks and in SD_1_ only in Mallard ducks (Table 6). Regarding the thigh meat quality of Muscovy ducks, it was the best in SD_1_ and SD_2_ when compared with SD_3_, but there was no significant difference between SD_1_ and SD_2_. While, in Mallard ducks, the best quality was recorded for SD_1_ when compared with SD_2_ and SD_3_. Moreover, SD_2_ possessed a higher quality than that of SD_3_ (Table 7).

**Table 6:** Meat quality of pectoral major muscle of ducks reared with different stocking density.

	Muscovy breed					Mallard breed				
Parameters	SD_1_	SD_2_	SD_3_	SEM	*P-*value	SD_1_	SD_2_	SD_3_	SEM	*P-*value
pH_u_	6.07^a^	5.96^a^	5.58^b^	0.07	0,001	6.13^a^	6.03^a^	5.67^b^	0.06	0,000
Cooking loss %	24.98^b^	25.97^b^	31.24^a^	0.68	0,000	10.08^c^	11.06^b^	12.04^a^	0.22	0,000
Drip loss %	3.96^b^	4.12^b^	5.48^a^	0.17	0,000	1.77^c^	3.00^b^	5.66^a^	0.38	0,000
Total bacterial count (Log CFU/g)*	4.70^b^	4.69^b^	5.05^a^	0.05	0,001	3.65^c^	3.78^b^	3.95^a^	0.03	0,000
Enterobacteriaceae (Log CFU/g)*	3.01^b^	3.00^b^	3.57^a^	0.06	0,000	3.02^b^	3.03^b^	3.48^a^	0.05	0,000
Coliforms bacteria (Log CFU/g)*	2.49^b^	2.50^b^	3.22^a^	0.09	0,000	2.49^c^	2.65^b^	3.12^a^	0.06	0,000

SD_1_: stocking density of 5 ducks/m^2^; SD_2_: stocking density of 7 ducks/m^2^; SD_3_: stocking density of 9 ducks/m^2^.

SEM, standard error mean.

a,b,c Means within the same row with different superscripts letter was differ significantly.

⁎ Results of this table were of Log transformed data.

**Table 7:** Meat quality of thigh muscle of ducks reared with different stocking density.

	Muscovy breed					Mallard breed				
Parameters	SD_1_	SD_2_	SD_3_	SEM	*P-value*	SD_1_	SD_2_	SD_3_	SEM	*P-*value
pH_u_	6.12^a^	6.06^a^	5.52^b^	0.08	0,000	6.13^a^	6.04^b^	6.06^b^	0.01	0,002
Cooking loss	31.30^b^	32.28^b^	35.32^a^	0.43	0,000	15.17^c^	16.51^b^	18.02^a^	0.27	0,000
Drip loss	2.024^c^	2.59^b^	3.38^a^	0.13	0,000	1.73^c^	3.62^b^	6.07^a^	0.40	0,000
Total bacterial count (Log CFU/g)*	4.90^b^	4.94^b^	5.17^a^	0.03	0,000	3.77^c^	3.93^b^	4.24^a^	0.05	0,000
Enterobacteriaceae (Log CFU/g)*	3.01^b^	3.09^b^	3.72^a^	0.08	0,000	3.01^c^	3.17^b^	3.73^a^	0.07	0,000
Coliforms bacteria (Log CFU/g)*	2.59^b^	2.78^b^	3.82^a^	0.14	0,000	2.59^c^	3.41^b^	3.79^a^	0.11	0,000

SD_1_: stocking density of 5 ducks/m^2^; SD_2_: stocking density of 7 ducks/m^2^; SD_3_: stocking density of 9 ducks/m^2^.

SEM, standard error mean.

a,b,c Means within the same row with different superscripts letter was differ significantly.

⁎ Results of this table were of Log transformed data.

## DISCUSSION

This study explored if SDs influence on growth, carcass parameters, blood indices, and meat quality of Muscovy and Mallard duck to advocate good SD alongside cheapest coast concurrently with superior property. This study revealed that the body weight and weight gain of both duck breeds were reduced by increasing SD. These results were supported by the finding of other researchers on ducks (Xie et al., 2014; Wu et al., 2018; Zhang et al., 2018) and broilers (Nasr et al., 2021), in spite of keeping the same feeding area for each bird (Sørensen et al., 2000; Tong et al., 2012). Moreover, Taboosha (2014) detected that ducks raised at SD of 5 ducks/m^2^ possessed the highest body weight and body weight gain than densities of 7 ducks/m^2^ which supported our findings. The body weight in this study was similar to that reported by Hassan, et al. (2018), who stated that the final body weight of Muscovy duck was 3,904 g at age of 84 d, while for Mallard ducks was 4,021 g at age of 76 d (Omar et al., 2019). On contrast, stoking densities did not affect the final live weight (Gupta et al., 2016).

The reduction of body weight and body weight gain in high SD might be retained to numerous suggestions: 1) decreased area for each birds, consequently forced the birds to stand and increased their energy requirements and decreased the ability to rest (Zulkifli and Siti Nor Azah, 2004; Buijs et al., 2010), 2) changes of gut morphology (Li et al., 2017), 3) chronic oxidative stress (Simitzis et al., 2012), 3) high temperature stress of the birds per unit, 4) inadequate air exchange, 5) high ammonia, 6) decreased diet palatability and drinking water (Simsek et al., 2009), 7) high levels of CO2 and NH3 in the pen (Guardia et al., 2011; Petek et al., 2014; Van Staaveren et al., 2019). Moreover, high stocking densities deteriorated the duck's antioxidant ability, consequently interrupt the antioxidant defense system and finally caused oxidative stress (Simsek et al., 2009; Zhang et al., 2015). High SD interrupt the heat emission to air to help ducks for intensive growth, that influenced by the large group size even with keeping the same feeding area for each bird (Sørensen et al., 2000; Tong et al., 2012).

The carcass traits are an imperative economic aspect of the poultry business (Nasr et al., 2017, 2019). This investigation revealed that low SD (5 ducks/m^2^) had the best carcass traits of both duck breeds. The findings of this study were supported with researchers who stated that high SD changes the carcass parameters and lowered the carcass property (Skomorucha et al., 2009; Sekeroglu et al., 2011) decreased breast fillet, whole breast yield and thigh (Wu et al., 2018; Abo Ghanima et al., 2020). Moreover, the meat traits decreased with increasing SD to 9 birds/m^2^ (Xie et al., 2014). This reduction and deterioration of carcass traits may be due to the high SD caused physical constraint of birds movement, consequently affect birds to reach to the feed and water (SCAHAW Scientific Committee on Animal Health and Animal Welfare, 2000). On the other hand, other researchers stated no effect of SD on carcass traits, breast, and leg weight and dressing % of Muscovy ducks (Baeza et al., 2003; Xie et al., 2014; Gupta et al., 2016), even with stoking densities of 5, 6, and 7 birds/m^2^ (Taboosha, 2014).

Different stocking densities had no effect on gizzard and liver weight of both duck breeds which supported by Bawish (2018) and confirmed by Ahaotu and Agbasu (2015) who mentioned that pekin ducks densities of 2, 4, 6, and 8 ducks/m^2^ had no effects on giblets weight. While, our findings regarding the spleen, revealed an increase of its weight that supported by others (Chegini et al., 2018). They detected that the spleen weight was enlarged in high SD. This may be attributable to the stress that may increase the lymphoid organ to promote the immune system (Gore and Qureshi, 1997; Pope, 1991).

Blood biochemical parameters are decisive tool for predicting the metabolic diseases (Rotava et al., 2008). Stress caused an alteration of birds' blood caused by the thermoregulatory reactions (Arieli et al., 1979). High SD has a negative consequence on birds' performance and physiological traits (Cengiz et al., 2015). Corticosterone is of little useful guide chronic stress (Cunningham et al., 1988), while heterophil/lymphocyte (**H/L**) ratio is counted as a marker of chronic stress in birds (Gross and Siegel, 1983; Zulkifli et al., 2003) and hens welfare (Nicol et al., 2009). There were arguing conclusions on the impact of SD on ducks' H/L ratio. Several studies reported an increase of this ratio with increasing density (Hill, 1983; Stevenson and Taylor, 1988; De Jong et al., 2002; Srinongkote et al., 2004). However, other researchers did not detect any effect (Dozier et al., 2006; Turkyilmaz, 2008). The current investigation was similar to the majority of researches that detected an increase of H/L ratio as a result of increasing density. Our findings regarding Heterophil and lymphocyte were comparable with Abdel-Rahman and Mosaad (2013). Otherwise, Gupta et al. (2016) did not detect any impact of high SD on Heterophil/lymphocyte ratio.

It was no variation regarding serum total protein, albumin, globulin, and A/G ratio of both duck breeds at different stocking densities. These findings were supported by the findings of other researchers (Abu-Tabeekh, 2013; Azzam and El-Gogary, 2015; Bawish, 2018). Otherwise, it was disagreed with Srinongkote et al. (2004) and Nasr et al. (2021), who detected an increase of the serum protein with increasing density. Our values of serum total protein, albumin, and globulin were similar to that reported by Abdel-Rahman and Mosaad (2013) for Muscovy ducks.

Abo-Ghanima et al. (2020) and Park et al. (2018) reported an increase of ALT and AST with increasing the duck density from 3 to 7 ducks/m^2^. All these results were confirmed the current findings that ALT and AST increased with high density of both duck breeds. High SD may cause heat-stressed ducks that consequently elevates the AST and ALT (Park et al., 2018). The disruptions in liver enzyme activities may be related to the disorder in body homeostasis due to the high SD, accordingly birds will attempt to become accustomed themselves to the stressors through behavioral and physiological adjustments (Bueno et al., 2017). Moreover, birds' struggle to consume more feed at high densities that will prone to muscles damage and finally increased AST and ALT in the blood.

There was no significant difference of cholesterol, triglycerides and HDL of Muscovy ducks at different densities. These findings were comparable to other researchers (Abu-Tabeekh, 2013; Bawish, 2018; Mallick et al., 2018). While, they were increased with increasing densities in Mallard ducks, which was in accordance with Wu, et al. (2018), who detected an elevation of cholesterol and triglycerides at high density of Peking ducks (9/m^2^). Moreover, cholesterol, triglycerides, and LDL were highest in high density (6 birds/m^−2^) when compared with 3 and 4 birds m^−2^ (Park et al., 2018).

In this study high density prompted oxidative stress, since its increased MDA level and declined the activity of serum SOD and GPx. The current finding was fortified by preponderance of studies that inspecting the destructive impact of high SD in birds (Simitzis et al., 2012; Zhang et al., 2015; Liu et al., 2016; Abo Ghanima et al., 2020). This may be owing to the overcrowding that encouraging fights between animals and initiating metabolic disorders. Moreover, prompting stress, elevated lipid peroxidation and excessive creation of ROS, raised oxidative damage and producing MDA as a result of reducing the performance of antioxidant enzymes (Droge, 2002; Yun-Zhong et al., 2002; Simsek et al., 2009).

Complement 3 and IgG reduced at high stoking density of both duck breeds that confirmed by others (Mashaly et al., 2004; Palizdar et al., 2017). They reported high densities smoothers the immunity (Zhang et al., 2015). Liberating somatostatin and adrenal corticosteroid hormones were accountable for reducing immunoglobulin synthesis (Herman et al., 2018). In contrast, there was a rise of IgG and IgM at higher densities (Li et al., 2019), while Azzam and El-Gogary (2015) did not observed any impact of SD on immunoglobulin. High densities accelerated the physiological and oxidative stress and exhilarated intestinal mucosal destruction. Thus, they were vulnerable to infectious diseases (Li et al., 2019).

Ultimate muscle pH (pHu) is an imperative guide of muscle glycolysis, meat property and muscle acidity that affect the colour, tenderness and meat storage time. Muscle lactic acid are a sign of pH and the quality of meat decreased with their high accumulations (Chen et al., 2015). Meat pHu of Muscovy and Mallard duck breeds (thigh and breast) is ranged between 5.7 and 6.17 (Wawro et al., 2004; Chen et al., 2015). Moreover, meat pH of more than 5.8 is preferable to increase meat shelf life (Ali et al., 2007). Our finding regarding pHu of both ducks breeds in breast and thigh were within this range. Also, pHu levels decreased with increasing SD. The reduction of pH accelerates the anaerobic glycolysis and lactate accumulation (Rammouz et al., 2004). A prompt decrease of pH in early postmortem stage initiates extensive protein denaturation (Huff-Lonergan and Lonergan, 2005). On contrast, there was no significant effect of stocking densities on pHu and cooking loss (Xie et al., 2014; Zhang et al., 2018).

Drip loss is a popular indirect marker of water-holding capacity, since water loss is negatively interrelated with water-holding capacity (Chen et al., 2015). The effect of high SD on cooking loss and drip loss was more obvious in Mallard ducks than Muscovey ducks. Also, with high densities they were increased and this was confirmed by Zhang et al. (2018) who reported a reduction of drip loss by increasing SD. Zhang et al. (2018) stated that the drip loss was increased with increasing density from 5 to 8 and 11 birds/m^2^ of the ducks’ breast muscle. These findings suggested that meat is further vulnerable to turn into dry, hard and may be tasteless. Omojola (2007) detected that the cooking loss was 26 to 32% for Muscovy duck gave that was comparable to our findings of Muscovy ducks. Deterioration of meat quality could be related to decrease the competence of myofibrillar protein to grasp water owned to disturb collagen and myofibrillar protein matrix. This has been carried out throughout the ageing and water pressed out from myofibrils to channels that located among muscle fiber and cell membrane. Hence the contraction at rigor mortis leads to water could leave as drip (Lawson, 2004). Low pH stimulates muscle fiber contraction, causing high drip loss (Tang et al., 2013). The more drip loss was steady with the low pH that causes inferior meat quality of ducks.

High densities are accompanying with confrontational effects on the birds' intestinal commensal bacteria (Cengiz et al., 2015). Additionally, stress may change the leucocytes and relegated the humoral immunity, accordingly, diminish the birds immunity to overwhelmed the infections with bacteria and viruses (Mench et al., 1986). High SD revealed the greatest bacterial and infection of breast and thigh muscles of both ducks' breed. Our outcomes regarding Enterobacteriaceae counts are within the reported range of 2.2 to 3.8 (log _10_ cfu/g) in ducks' breast muscle (Khalifa and Nassar, 2001). But, was lower than detected by Abdallaha, et al. (2014). They reported that Enterobacteriaceae and Coliform counts in ducks' breast muscle were 7.85 × 10^3^ and 1.70 × 10^2^ cfu/g, respectively while in thigh muscle were 9.13 × 10^4^ and 3.29 × 10^2^ cfu/g, respectively. Birds' litter is a mixture of birds' dropping and bedding stuffs, which deemed an ecological ecosystem with existence of microbial infection (Lovanh et al., 2007). Bacterial growth was increased in presence of high SD as a result of increasing the levels of ammonia and moisture in the litter, spilled water out and unsatisfactory ventilation, thus downgrading the litter quality. SD is deemed to be one of the foremost aspects affecting poultry welfare, physical performance, and product excellence. This investigation accomplished that high SD has an unfavorable consequence on ducks’ growth and welfare. High density revealed the smallest body weight, carcass traits, HDL, GPX, and IGG with great values of ALT, AST, MDA, cooking loss, drip loss of breast and thigh muscles, and bacterial count of both breeds. For that reason, this investigation suggested that the best density of Muscovy and Mallard breed is SD_1_ (5 birds/m^2^).

## References

[bib0001] Abdallaha R.N., Hassanen F.S., Salem A.M., El-Shater M.A.H. (2014). Bacterial evalution of frozen cut-up duck meat. Behav. Vet. Med. J..

[bib0003] Abdel-Rahman M.A., Mosaad G.M. (2013). Effect of propolis as additive on some behavioural patterns, performance and blood parameters in Muscovy broiler ducks. J. Adv. Vet. Res..

[bib0004] Abo-Ghanima M.M., Abd El-Hack M.E., Taha A.E., Tufarelli V., Laudadio V., Naiel M.A.E. (2020). Assessment of stocking rate and housing system on performance, carcass traits, blood indices, and meat quality of French Pekin Ducks. Agriculture.

[bib0006] Abu- Tabeekh M.A.S. (2013). The effect of color light and stocking density on some biochemical traits of broilers and layers. Res. Zool..

[bib0008] Ahaotu E.O., Agbasu C.A. (2015). Evaluation of the stocking rate on growth performance, carcass traits and meat quality of male Pekin ducks. Sci. J. Biol. Sci..

[bib0009] Ali M.S., Kang G.H., Yang H.S., Jeong J.Y., Hwang Y.H., Park G.B., Joo S.T. (2007). A comparison of meat characteristics between duck and chicken breast. Asian-Aust. J. Anim. Sci..

[bib0011] (2016).

[bib0012] Arieli A., Meltzer A., Berman A. (1979). Seasonal acclimatization in the hen. Br. Poult. Sci..

[bib0013] Azzam M.M.M., El-Gogary M.R. (2015). Effects of dietary threonine levels and stocking density on the performance, metabolic status and immunity of broiler chickens. Asian J. Anim. Vet. Adv..

[bib0014] Baeza E., Chartrin P., Arnould C. (2003). Effect of stocking density on welfare, growth performance and carcass quality in Muscovy ducks. Sci. Tech. Avi..

[bib0015] Baeza E., Salichon M.R., Marche G., Wacrenier N., Dominguez B., Culioli J. (2000). Effect of age and sex on the structural, chemical, technological characteristics of mule duck meat. Br. Poult. Sci..

[bib0017] Bawish, B.M., 2018. Effect of management factors on welfare and productive performance of muscovy ducks. Ph.D. Thesis in Veterinary Medicine Science. 2018, Cairo University, Faculty of Veterinary Medicine, Veterinary Hygiene and Management, Cairo, Egypt

[bib0019] Berg, C., and J. Yngvesson. 2012. Optimal stocking density for broilers optimal for whom? In XXIVWorld Poultry Congress, Area: Poultry Welfare and Environment 5–9 August, Salvador, Bahia, Brazil.

[bib0020] Bueno J.P.R., Nascimento M.R.B., Martins J.M., Marchini C.F.P., Gotardo L.R.M., Sousa G.M.R., Mundim A.V., Guimarães E.C., Rinaldi F.P. (2017). Effect of age and cyclical heat stress on the serum biochemical profile of broiler chickens. Semin. Ciências Agr. Londrina.

[bib0021] Buijs S., Keeling L.J., Vangestel C., Baert J., Vangeyte J., Tuyttens F.A.M. (2010). Resting or hiding? Why broiler chickens stay near walls and how density affects this. Appl. Anim. Behav. Sci..

[bib0022] Cengiz O.K., Oksal B.H., Tatlı O., Sevim O., Ahsan U., Uner A.G., Uluta P.A., Beyaz D., Buyukyoruk S., Yakan A., Onol A.G (2015). Effect of dietary probiotic and high stocking density on the performance, carcass yield, gut microflora, and stress indicators of broilers. Poult. Sci..

[bib0023] Chegini S., Kiani A., Rokni H. (2018). Alleviation of thermal and overcrowding stress in finishing broilers by dietary propolis. Italian J. Anim. Sci..

[bib0024] Chen Y., Aorigele C., Yan F., Li Y., Cheng P., Qi Z. (2015). Effect of production system on welfare traits, growth performance and meat quality of ducks. South Afr. J. Anim. Sci..

[bib0025] Cherry P., Morris T.R. (2015). Page 239 in Science and Practice.

[bib0026] Crawford R.D., Crawford R.D. (1990). Pages 1–41 in Poultry Breeding and Genetics.

[bib0027] Cunningham D.L., van Tienhoven A., Gvaryahu G. (1988). Population size, cage area, and dominance rank effects on productivity and well-being of laying hens. Poult. Sci..

[bib0028] Cyril H.W., Castellini C., Dal Bosco A. (1996). Comparison of three cooking methods of rabbit meat. Italian J. Food Sci..

[bib0029] De Jong I.C., Van Voorst S., Ehlhardt D.A., Blokhuis H.J. (2002). Effects of restricted feeding on physiological stress parameters in growing broiler breeders. Br. Poult. Sci..

[bib0030] Dozier W., Thaxton J., Purswell J., Olanrewaju H., Branton S., Roush W. (2006). Stocking density effects on male broilers grown to 1.8 kilograms of body weight. Poult. Sci..

[bib0031] Dröge W. (2002). Aging-related changes in the thiol/disulfide redox state:implications for the use of thiol antioxidants. Exp. Gerontol.

[bib0032] Ekarius C. (2007).

[bib0033] Etches, R. J., T. M. John, and A. M. V. Gibbins. 2008. Behavioural, physiological, neuroendocrine and molecular responses to heat stress, in: Poultry production in hot climates, edited by: N. J. Daghir, Trowbridge, Cromwell press. 49–80.

[bib0034] Gore A.B., Qureshi M.A. (1997). Enhancement of humoral andcellular immunity by vitamin E after embryonic exposure. Poult. Sci..

[bib0035] Górski J., Witak B. (2003). Production results of white and black-and-white muscovy ducks fed with different feed mixtures. Ann. Univ. Mariae Curie-Sklodowska. Sec EE: Zootech..

[bib0036] Gross, W. B., H. S. Siegel. 1983. Evaluation of the Heterophil/Lymphocyte Ratio as a Measure of Stress in Chickens. Avian Disease, (p. 27), 972. 6360120

[bib0037] Guardia S., Konsak B., Combes S., Levenez F., Cauquil L., Guillot J.F., Moreau-Vauzelle C., Lessire M., Juin H., Gabriel I. (2011). Effects of stocking density on the growth performance and digestive microbiota of broiler chickens. Poult. Sci..

[bib0038] Gupta S.K., Behera K., Pradhan C.R., Acharya A. (2016). Influence of stocking density on the performance, carcass characteristics, hemato-biochemical indices of Vanaraja chicken. Indian J. Anim. Res.

[bib0039] Hassan F.A.M., Roushdy E.M., Zaglool A.W., Ali M.A., EL-Araby I.E. (2018). Growth performance, carcass traits and economic values of pekin, Muscovy, and Mullard ducks. Slovenian Vet. Res..

[bib0040] Herman J.P., Mueller N.K., Figueiredo H. (2018). Role of GABA and glutamate circuitry in hypothalamo-pituitary-adrenocortical stress integration. Ann. N. Y. Acad. Sci.

[bib0041] Hill J.A. (1983). Indicators of stress in Poultry. Worlds Poult. Sci. J..

[bib0042] Holderread D. (2011).

[bib0043] Houshmand M., Azhar K., Zulkifli I., Bejo M.H., Kamyab A. (2012). Effects of prebiotic, protein level, and stocking density on performance, immunity, and stress indicators of broilers. Poult. Sci..

[bib0044] Huff-Lonergan E., Lonergan S.M. (2005). Mechanisms of water holding capacity of meat: the role of postmortem biochemical and structural changes. Meat Sci..

[bib0045] Khalifa A.H., Nassar A.M. (2001). Nutritional and bacteriological of some game duck carcasses. Nahrung/Food.

[bib0046] Kilany W.H., Safwat M., Mohammed S.M., Salim A., Fasina F.O., Fasanmi O.G. (2016). Protective efficacy of recombinant turkey herpes virus (rHVT-H5) and inactivated H5N1 vaccines in commercial Mulard ducks against the highly pathogenic avian influenza (HPAI) H5N1 Clade 2.2.1 virus. PLoS One.

[bib0047] Korkeala H., Mäki-Petäys O., Alanko T., Sorvettula O. (1986). Determination of pH in meat. Meat Sci..

[bib0049] Lawson M.A. (2004). The role of integrin degradation in post-mortem drip loss in pork. Meat Sci..

[bib0051] Li J.H., Miao Z.Q., Tian W.X., Yang Y., Wang J.D., Yang Y. (2017). Effects of different rearing systems on growth, small intestinal morphology and selected indices of fermentation status in broilers. Anim. Sci. J..

[bib0052] Li X.M., Zhang M.H., Liu S.M., Feng J.H., Ma D.D., Liu Q.X., Zhou Y., Wang X.J., Xing S. (2019). Effects of stocking density on growth performance, growth regulatory factors, and endocrine hormones in broilers under appropriate environments. Poult. Sci..

[bib0053] Liu H., Rocha C.S., Dandekar S., Wan Y.J. (2016). Functional analysis of the relationship between intestinal microbiota and the expression of hepatic genes and pathways during the course of liver regeneration. J. Hepatol..

[bib0054] Lovanh N., Cook K.L., Rothrock M.J., Miles D.D.M., Sistani K. (2007). Spatial shifts in microbial population structure within poultry litter associated with physicochemical properties. Poult. Sci..

[bib0055] Mallick S.B., Mohanty G.P., Mishra S.K., Behera K., Dash S.K., Sethy K., Pati P.K., Mohapatra L.M. (2018). Performance study of white pekin ducks in different stocking density in floor rearing system. Indian J. Anim. Sci..

[bib0056] Mashaly M.M., Hendricks G.L., Kalama M.A., Gehad A.E., Abbas A.O., Patterson P.H. (2004). Effect of heat stress on production parameters and immune responses of commercial laying hens. Poult. Sci..

[bib0057] Mello J.L.M., Boiago M.M., Giampietro-Ganeco A., Berton M.P., Vieira L.D.C., Souza R.A., Ferrari F.B., Borba H. (2015). Periods of heat stress during the growing affects negatively the performance and carcass yield of broilers. Arch. Zootech..

[bib0058] Mench J.A., van-Tienhoven A., Marsh J.A., McCormick C.C., Cunningham D.L., Baker R.C. (1986). Effects of cage and floor pen management on behavior, production, and physiological stress responses of laying hens. Poult. Sci..

[bib0061] Nasr M.A.F., Ali E.M.R., Hussein M.A. (2017). Performance, carcass traits, meat quality and amino acid profile of different Japanese quails strains. J. Food Sci. Technol..

[bib0062] Nasr M.A.F., Alkhedaide A.Q., Ramadan A.A.I., Hafez A.E., Hussein M.A. (2021). Potential impact of stocking density on growth, carcass traits, indicators of biochemical and oxidative stress and meat quality of different broiler breeds. Poult. Sci..

[bib0063] Nasr M.A.F., Mohammed H., Hassan R.A., Swelum A.A., Saadeldin I.M. (2019). Does light intensity affect the behavior, welfare, performance, meat quality, amino acid profile, and egg quality of Japanese quails?. Poult. Sci..

[bib0064] National Research Council (NRC) (1994).

[bib0065] Nicol C.J., Caplen G., Edgar J., Browne W.J. (2009). Associations between welfare indicators and environmental choice in laying hens. Anim. Behav..

[bib0066] Oluyemi J.A., Ologbobo A.D. (1997). Pages 96–103 in Proceedings of the 2nd Annual Conference of the Animal Science Association of Nigeria.

[bib0067] Omar M.A., Abdel-hamid T.M., Esam S., Omar A.E. (2019). Growth and economic performance of using dried tomato pomace for mallard ducks. Slovenian Vet. Res..

[bib0068] Omojola A.B. (2007). Carcass and organoleptic characteristics of duck meat as influenced by breed and sex. Int. J. Poult. Sci..

[bib0069] Palizdar M.H., Daylami M.K., Pourelmi M.R. (2017). Effects of high stocking density on growth performance, blood metabolites and immune response of Broilers (ROSS 308). J. Livest. Sci..

[bib0070] Park B.S., Um K.H., Park S.O., Zammit V.A. (2018). Effect of stocking density on behavioral traits, blood biochemical parameters and immune responses in meat ducks exposed to heat stress. Arch. Anim. Breed.

[bib0071] Petek M., Üstüner H., Yesilba˘g D. (2014). Effects of stocking density and litter type on litter quality and growth performance of broiler chicken. Kafkas Univ. Vet. Fak. Derg.

[bib0072] Pingel H. (1999). Influence of breeding and management on the efficiency of duck production. Lohmann Inform.

[bib0073] Pingel H. (2004). Duck and geese production. World Poult..

[bib0074] Pope C.R. (1991). Pathology of lymphoid organs with emphasis onimmunosuppression. Vet. Immunol. Immunopathol..

[bib0075] Rammouz R.E., Babil´e R., Fernandez X. (2004). Effect of ultimate pH on the physicochemical and biochemical characteristics of turkey breast muscle showing normal rate of postmortem Ph fall. Poult. Sci..

[bib0076] Rotava R., Zanella I., Karkow A.K., Dullius A.P., da Silva L.P., Denardin C.C. (2008). Bioquímica sanguínea de frangos de corte alimentados com subprodutos da uva. Agrarian.

[bib0077] SAS (2009).

[bib0078] SCAHAW (Scientific Committee on Animal Health and Animal Welfare). (2000). The welfare of chickens kept for meat production (broilers). European Commission, Health and Consumer Protection Directorate- General. Accessed July 6, 2022. https://ec.europa.eu/food/system/files/2020-12/sci-com_scah_out39_en.pdf.

[bib0079] Sekeroglu A., Sarica M., Gulay M.S., Duman M. (2011). Effect of stocking density on chick performance, internal organ weights and blood parameters in broilers. J. Anim. Vet. Adv..

[bib0081] Shanawany M.M. (1988). Broiler performance under high stocking densities. Br. Poult. Sci..

[bib0083] Simitzis P., Kalogeraki E., Goliomytis M., Charismiadou M., Triantaphyllopoulos K., Ayoutanti A., Niforou K., Hager-Theodorides A., Deligeorgis S. (2012). Impact of stocking density on broiler growth performance, meat characteristics, behavioural components and indicators of physiological and oxidative stress. Br. Poult. Sci..

[bib0084] Simsek U., Cerci I., Dalkilic B., Yilmaz O., Ciftci M. (2009). Impact of stocking density and feeding regimen on broilers: chicken meat composition, fatty acids, and serum cholesterol levels. J. Appl. Poult. Res.

[bib0085] Skomorucha I., Muchacka R., Sosnowka-Czajka E., Herbut E. (2009). Response of broiler chickens from three genetic groups to different stocking densities. Ann. Anim. Sci..

[bib0086] Sørensen P., Su G., Kestin S.C. (2000). Effects of age and stocking density on leg weakness in broiler chickens. Poult. Sci..

[bib0087] Srinongkote S., Smriga M., Toride Y. (2004). Diet supplied with L-lysine and L-arginine during chronic stress of high stock density normalizes growth of broilers. Anim. Sci. J*.*.

[bib0088] Stevenson J.R., Taylor R. (1988). Effects of glucocorticoid and antiglucocorticoid hormones on leukocyte numbers and function. Int. J. Immunopharmacol..

[bib0089] Taboosha M.F. (2014). Effect of stocking density and slaughter age on growth performance and carcass traits of female mule ducks (crossbred of Muscovy Drake and Pekin Ducks) during summer season in Egypt. Middle East J. Appl. Sci..

[bib0090] Tang S., Zhang M., Bao E. (2013). Effects of different heat stress periods on various blood and meat quality parameters in young Arbor Acer broiler chickens. Can. J. Anim. Sci..

[bib0091] Tong H.B., Lu J., Zou J.M., Wang Q., Shi S.R. (2012). Effects of stocking density on growth performance, carcass yield, and immune status of a local chicken breed. Poult. Sci..

[bib0092] Turkyilmaz M.K. (2008). The effect of stocking density on stress reaction in broiler chickens during summer. Turk. J. Vet. Anim. Sci.

[bib0093] Vanhonacker F., Verbeke W., Van Poucke E., Buijs S., Tuyttens F.A.M. (2008). Societal concern related to stocking density, pen size and group size in farm animal production. Livest. Sci..

[bib0094] Van Staaveren N., Decina C., Baes C.F., Widowski T.M., Berke O., Harlander-Matauschek A. (2019). Housing and management practices on 33 pullet farms in Canada. Animals.

[bib0095] Wawro K., Wilkiewicz-Wawro E., Kleczek K., Brzozowski W. (2004). Slaughter value and meat quality of muscovy ducks, pekin ducks and their crossbreeds, and evaluation of the heterosis effect. Arch. Tierz. Dummerstorf.

[bib0096] Woloszyn J., Ksiazkiewicz J., Skrabka-Blotnicka T., Haraf G., Biernat J., Kisiel T. (2006). Comparison of amino acid and fatty acid composition of duck breast from five flocks. Arch. Tierzucht.

[bib0097] Wu Y., Li J., Qin X., Sun S., Xiao Z., Dong X., Shahid M.S., Yin D., Yuan J. (2018). Proteome and microbiota analysis reveals alterations of liver-gut axis under different stocking density of Peking ducks. PLoS One.

[bib0098] Xie M., Jiang Y., Tang J., Wen Z.G., Huang W., Hou S.S. (2014). Effect of stocking density on growth performance, carcass traits, and foot pad lesions of White Pekin ducks. Poult. Sci..

[bib0100] Yun-Zhong F., Sheng Y., Guoyao W. (2002). Regulation of physiological systems by nutrients: free radicals, antioxidants, and nutrition. Nutrition.

[bib0101] Zhang L., Zhang Y., Liu Y., Cai S., Yuan J., Wang Z. (2015). Effects of stocking density on immune function and oxidative stress level of Pekin Ducks reared on plastic wire-floor. China Poult..

[bib0103] Zhang Y.R., Zhang L.S., Wang Z., Liu Y., Li F.H., Yuan J.M., Zia Z.F. (2018). Effects of stocking density on growth performance, meat quality and tibia development of Pekin ducks. Anim. Sci. J..

[bib0104] Zulkifli I., Siti Nor Azah A. (2004). Fear and stress reactions and the performance of commercial broiler chickens subjected to regular pleasant and unpleasant contacts with human being. Appl. Anim. Behav. Sci..

[bib0105] Zulkifli I.P., Liew K., Israf D.A., Omar A.R., Hair-Bejo M. (2003). Effects of early age feed restriction and heat conditioning on heterophil/lymphocyte ratios, heat shock protein 70 expression and body temperature of heat-stressed broiler chickens. J. Ther. Biol..

